# Seasonality of Cytokines in Inflammatory Disease: Exploring Molecular Parallels with the Yellow Emperor’s Inner Canon

**DOI:** 10.3390/biology15181668

**Published:** 2026-09-20

**Authors:** Yang Liu, Ye Zeng, Bingmei M. Fu

**Affiliations:** 1 Institute of Biomedical Engineering, West China School of Basic Medical Sciences and Forensic Medicine, Sichuan University, Chengdu 610041, China; 2 The Hong Kong Polytechnic University, Hong Kong SAR, China; 3 Department of Biomedical Engineering, The City College of the City University of New York, New York, NY 10031, USA; fu@ccny.cuny.edu

**Keywords:** cytokine-mediated pathophysiology, inflammatory joint disease, LPS-mediated inflammation, seasonal disease progression, Yellow Emperor’s Inner Canon

## Abstract

Inflammatory cytokines are well-established drivers of organ-specific pathology, yet their seasonal patterning remains poorly integrated into a single explanatory framework. This review examines whether classical descriptions of Bi syndrome in the Yellow Emperor’s Inner Canon (YEIC), translated using previously proposed molecular correspondences (Cold/IL-1, Dampness/IL-6, Wind/NF-κB, Evil/LPS) and treated here as a working hypothesis, are consistent with the contemporary biomedical literature. For each YEIC season-tissue pairing, relevant evidence was retrieved from PubMed covering seasonal disease epidemiology, cytokine mechanisms, and clinical progression; correspondence with the YEIC’s descriptions was assessed qualitatively across five seasonal domains rather than through a literature review with predefined inclusion criteria. IL-1, IL-6, and NF-κB predominance was mapped thematically onto seasonal cycles of tissue involvement—bone (winter), tendons (spring), vasculature (summer), muscle (late summer), and skin (autumn)—with proposed progression to related visceral injuries. This correspondence is imperfect in at least one domain: cardiovascular disease shows a larger winter, rather than summer, peak in the broader literature, and the framework has not been tested against competing explanations such as temperature, vitamin D status, infection cycles, or behavior. These findings suggest, without establishing, that IL-1, IL-6, and NF-κB may offer a shared explanatory thread across conditions conventionally treated as etiologically distinct. The YEIC, reinterpreted through a molecular lens, offers a hypothesis-generating model requiring prospective, comparative validation.

## Simple Summary

This study examines whether a 2000-year-old Chinese medical textbook, the Yellow Emperor’s Inner Canon (YEIC), describes seasonal patterns of inflammatory diseases that align with modern medical observations. The researchers translated the ancient concepts of Cold, Dampness, and Wind into modern molecular terms corresponding to specific inflammatory cytokines: Interleukin-1, Interleukin-6, and Nuclear Factor kappa B. By comparing the YEIC’s theoretical pathways with current medical research, the study found a strong alignment: winter bone disorders theoretically progress to kidney problems, spring tendon issues to liver disease, summer blood vessel problems to heart conditions, late summer muscle pain to spleen disorders, and autumn skin conditions to lung problems. The ancient text also mapped these distinct inflammatory cytokines to different characteristics of joint pain. Rather than proving direct cause and effect, these findings propose a theoretical framework suggesting that conventionally irrelevant seasonal diseases may share interconnected inflammatory origins. This perspective serves to generate new hypotheses, inspiring future scientific investigation into the seasonal nature of human inflammation.

## 1. Background

Interleukin-1 (IL-1), Interleukin-6 (IL-6), and NF-κB are among the most extensively studied inflammatory mediators in modern medicine, implicated in a remarkably wide range of conditions—including skeletal disorders, acute kidney injury (AKI), tendinopathy, viral hepatitis, vascular disease, heart disease, myalgia, splenomegaly, eczema, and asthma. Despite shared molecular origins, these conditions are conventionally treated as independent diseases with distinct etiologies, and the deeper inflammatory connections among them have yet to be systematically recognized or emphasized in clinical practice. 

Notably, the Yellow Emperor’s Inner Canon (YEIC)—a foundational Chinese medical text compiled over two thousand years ago—describes with remarkable specificity what appears to be the same cluster of inflammatory diseases, their seasonal onset patterns, and their pathological progression from one organ/tissue system to another. That a pre-modern text could anticipate relationships that contemporary biomedicine has only partially recognized raises a question worth taking seriously: what did its authors observe, and what can modern medicine learn from it? 

The YEIC stands as one of the most systematic and enduring medical texts in human history. Compiled during the Axial Age (approximately 800–200 BCE)—a period Karl Jaspers [1] identified as one of the most consequential intellectual epochs in human history, whose insights “mankind is still living by”—it represents one of the most systematic attempts in the ancient world to describe human physiology and disease. Structured as dialogues between the legendary Yellow Emperor and a series of Wise Men, its two books—the Su Wen (“Plain Questions”) and the Ling Shu (“Spiritual Pivot”)—encompass 162 chapters covering vital substances, the meridian-collateral system, disease etiology, and therapeutic principles. For millennia, it served as the theoretical foundation of East Asian medicine, and it remains a canonical reference in Traditional Chinese Medicine (TCM) today. 

Modern medicine has viewed the YEIC with understandable skepticism on two grounds. First, its core physiological concepts—Qi, Cold, Dampness, Wind, Evil—are formulated in abstract terms with no obvious correspondence to concrete biochemical entities. Second, while the YEIC explicitly identifies meridians with the vascular system, the meridian pathways it describes differ substantially from modern cardiovascular anatomy. Some scholars have sought to rescue the text through metaphysical interpretation, but this has tended to deepen rather than resolve doubts about its scientific basis.

Rather than treating the YEIC’s terminology as inherently non-scientific, an alternative approach—proposed by the authors in prior work [2]—is to interpret its Vital Substances as systematically corresponding to specific modern signaling molecules. In this prior work, TCM Vital Substances were mapped onto candidate signaling molecules through a three-step approach. First, the physiological functions and interrelationships attributed to each vital substance under the YEIC’s “static” descriptions—those independent of time or location—were compiled across the text as a set of defining criteria. Second, a candidate molecule was considered a match if its own physiological functions, and its position within known signaling pathways, were consistent with these criteria. Third, each proposed mapping was further compared by testing whether classical TCM herbal medicines reported to regulate the vital substance produced comparable effects on the corresponding molecule. This approach yielded proposed correspondences for 48 TCM vital substances, of which Qi, Cold, Dampness, Wind, and Evil—mapped to sodium ions, IL-1, IL-6, NF-κB, and LPS, respectively, as shown in Table 1—form the basis of the present analysis. This molecular reinterpretation has since been applied to specific diseases. In a study of TCM’s understanding of diabetes (“Consumptive Thirsty” syndrome), Liu et al. [3] identified FoxO1 as the key molecular target underlying the TCM’s three-stage disease model, and showed that classical herbal formulas modulate FoxO1 activity in ways consistent with contemporary diabetes management. The meridian system, understood through this framework as describing sodium ion dynamics rather than blood circulation—a scale difference of roughly 80,000-fold—will be addressed in detail in a separate publication [4]. 

This molecular framework opens a new avenue for evaluating the YEIC’s clinical observations. The text describes Bi syndrome—caused by Cold, Dampness, and Wind—in terms that, translated into modern language, correspond to diseases mediated by IL-1, IL-6, and NF-κB. Crucially, the YEIC does not treat these as isolated conditions. It specifies their seasonal onset: skeletal disorders and kidney disease in winter, fascial and liver disorders in spring, vascular and heart disease in summer, skin and lung disease in autumn, and spleen disorders in late summer (July). It further describes predictable progression pathways: untreated skeletal disease advances to kidney injury, fascial disease to hepatitis, vascular disease to heart failure, muscle disease to spleen disorders, and skin disease to pulmonary involvement. 

Seasonal variation in immune activity has been demonstrated by genome-wide studies. Dopico et al. reported widespread seasonal transcriptional changes in human immune cells, including a winter-associated pro-inflammatory profile with increased soluble IL-6 receptor and C-reactive protein levels [5]. While seasonal variations in inflammatory diseases are well-documented, they are frequently attributed to parallel environmental factors such as temperature changes, circadian and circannual immunology, and behavioral shifts. This review aims to explore an alternative, unified theoretical framework derived from the Yellow Emperor’s Inner Canon (YEIC). We systematically evaluate whether the seasonal and organ-specific disease patterns described in the YEIC correspond to contemporary biomedical evidence on cytokine-mediated inflammatory pathophysiology, specifically IL-1, IL-6, and NF-κB.

## 2. Method

This study is a systematic analysis aimed at evaluating whether the seasonal and organ-specific disease patterns described in the Yellow Emperor’s Inner Canon (YEIC) correspond to contemporary biomedical evidence on cytokine-mediated inflammatory pathophysiology.

No human participants or experimental materials were involved. The primary source material comprised Chapter 43 and Chapter 55 of the Su Wen Book, drawn from the YEIC. Contemporary biomedical evidence was drawn from peer-reviewed literature retrieved from PubMed, covering epidemiological studies on seasonal disease incidence, molecular studies on cytokine mechanisms, and clinical reports on disease progression and manifestation.

The analytical framework proceeded in three steps. First, the YEIC’s descriptions of Bi syndrome—including its seasonal classifications, their cytokine equivalents (Cold/IL-1, Dampness/IL-6, Wind/NF-κB, Evil/LPS), and organ progression pathways—were extracted and translated into contemporary biomedical terminology based on the molecular correspondences proposed by the authors in prior work [2], which are treated here as a working hypothesis rather than an established finding. Second, for each season-tissue pair and organ progression pathway described in the YEIC, targeted literature searches were conducted to identify biomedical findings relevant to each proposed correspondence. Because the searches were organized around a pre-existing theoretical framework, they were not designed to establish causal validity or exclude alternative explanations. Therefore, the literature synthesis should be interpreted as an assessment of biological compatibility—a necessary but not sufficient step toward establishing the framework’s validity—rather than empirical validation of the proposed YEIC-molecular mappings. Third, the correspondence between YEIC descriptions and contemporary findings was assessed qualitatively across five seasonal domains: winter/bone-kidney, spring/tendon-liver, summer/vessel-heart, late summer/muscle-spleen, and autumn/skin-lung. 

As this is a systematic analysis based on existing published literature, no original data were collected, no statistical analysis was performed, and no power calculation was applicable.

### 2.1. Original Descriptions of Bi Syndrome in YEIC

Below, we present the description of Bi syndrome from Chapter 43 of Su Wen in the YEIC, with additional symptom descriptions from Chapter 55 incorporated in parentheses: 

The Yellow Emperor asked: How does Bi syndrome arise? 

QiBo answered: When the three pathogenic factors—Cold, Dampness and Wind—invade the body simultaneously, they form Bi syndrome. When Wind predominates, it is called “Migratory Bi”; when Cold predominates, it is called “Painful Bi”; when Dampness predominates, it is called “Fixed Bi”.

The Yellow Emperor further asked: What about the five types of Bi syndrome? QiBo said: When these pathogenic factors are contracted in winter, it causes “Bone Bi,” (characterized by heaviness of the bones, difficulty lifting the limbs, severe aching pain deep in the bone marrow, and a sensation of cold invasion); in spring, it causes “Tendon Bi,” (characterized by tendon spasms, joint pain, and difficulty walking); in summer, it causes “Vessel Bi”; in late summer (the transition between summer and autumn), it causes “Muscle Bi,” (characterized by pain throughout the body’s musculature); and in autumn, it causes “Skin Bi.”

The Yellow Emperor asked: What causes the pathogenic factors to invade the five Zang organs (including the liver, heart, spleen, lung, and kidney) and six Fu organs (including the stomach, large intestine, small intestine, bladder, gallbladder, and triple energizer)? QiBo said: Each of the five Zang organs corresponds to specific external tissues. If Bi syndrome persists without resolution, the pathogenic factors will penetrate deeper to invade the corresponding internal organs. Therefore, if Bone Bi does not resolve and the body is also exposed to Evil, they will invade the Kidneys; if Tendon Bi persists and the body is also exposed to Evil, they will invade the Liver; if Vessel Bi persists and the body is also exposed to Evil, they will invade the Heart; if Muscle Bi persists and the body is also exposed to Evil, they will invade the Spleen; if Skin Bi persists and the body is also exposed to Evil, they will invade the Lungs. All Bi syndromes result from repeated exposure to Wind, Cold, and Dampness during their respective seasons.

When Bi syndrome invades the five Zang organs: in Lung Bi, patients experience anxiety and chest tightness, short of breath, and experience vomiting; in Heart Bi, poor blood circulation, irritability accompanied by palpitations, dyspnea, dry throat, frequent belching, and feeling of fear; in Liver Bi, patients startle easily during sleep at night, experience excessive thirst with frequent drinking and urination, and abdominal distention similar to pregnancy; in Kidney Bi, patients experience abdominal distension, severe kyphosis (using the buttocks in place of the heels when standing), and inability to lift the head (using the spine in place of the head); in Spleen Bi, patients experience weakened limbs, coughing, vomiting liquid, and difficulty breathing.

### 2.2. Summary in Modern Languages

In this passage from the YEIC, “Cold” corresponds to IL-1, “Dampness” corresponds to IL-6, “Wind” corresponds to NF-κB, and “Evil” corresponds to LPS, depicting the effects of IL-1, IL-6, and NF-κB on the human body across different seasons, as well as the developmental pathways of diseases they trigger. Below, we summarize this passage in modern medical terminology, arranged by season.

1. When IL-1, IL-6, and NF-κB simultaneously invade the body, they form Bi syndrome. The predominant factor determines the presentation: NF-κB predominance produces “Migratory Bi” (migratory arthritis pain), IL-1 predominance produces “Painful Bi” (gout), and IL-6 predominance produces “Fixed Bi” (localized pain).
2. In winter, invasion causes “Bone Bi,” characterized by heaviness in the bones, difficulty lifting the limbs, severe aching pain deep within the bone marrow, and sensations of cold penetration. If Bone Bi persists and the body is additionally exposed to LPS, the pathogenic factors invade the Kidneys, resulting in Kidney Bi. Patients with Kidney Bi experience abdominal distension and severe kyphosis.
3. In spring, invasion causes “Tendon Bi,” characterized by tendon spasms, joint pain, and difficulty walking. If Tendon Bi persists with concurrent LPS exposure, the pathogenic factors invade the Liver, resulting in Liver Bi. Patients with Liver Bi startle easily during sleep, experience excessive thirst with frequent drinking and urination, and develop abdominal distention resembling pregnancy.
4. In summer, invasion causes “Vessel Bi.” If Vessel Bi persists with concurrent LPS exposure, the pathogenic factors invade the Heart, resulting in Heart Bi. Patients with Heart Bi experience poor circulation, irritability with palpitations, dyspnea, dry throat, frequent belching, and feelings of fear.
5. In late summer (July), invasion causes “Muscle Bi,” characterized by pain throughout the body’s musculature. If Muscle Bi persists with concurrent LPS exposure, the pathogenic factors invade the Spleen, resulting in Spleen Bi. Patients with Spleen Bi experience weakened limbs, coughing, vomiting of liquid, and difficulty breathing.
6. In autumn, invasion causes “Skin Bi.” If Skin Bi persists with concurrent LPS exposure, the pathogenic factors invade the Lungs, resulting in Lung Bi. Patients with Lung Bi experience anxiety, chest tightness, shortness of breath, and vomiting.

Below, we will compare the above content with modern medicine point by point. 

## 3. Results: Comparative Analysis of YEIC Descriptions and Contemporary Biomedical Findings

### 3.1. Bi Patterns by Cytokine: Migratory (NF-κB), Painful (IL-1), and Fixed (IL-6) 

**Figure 1:**
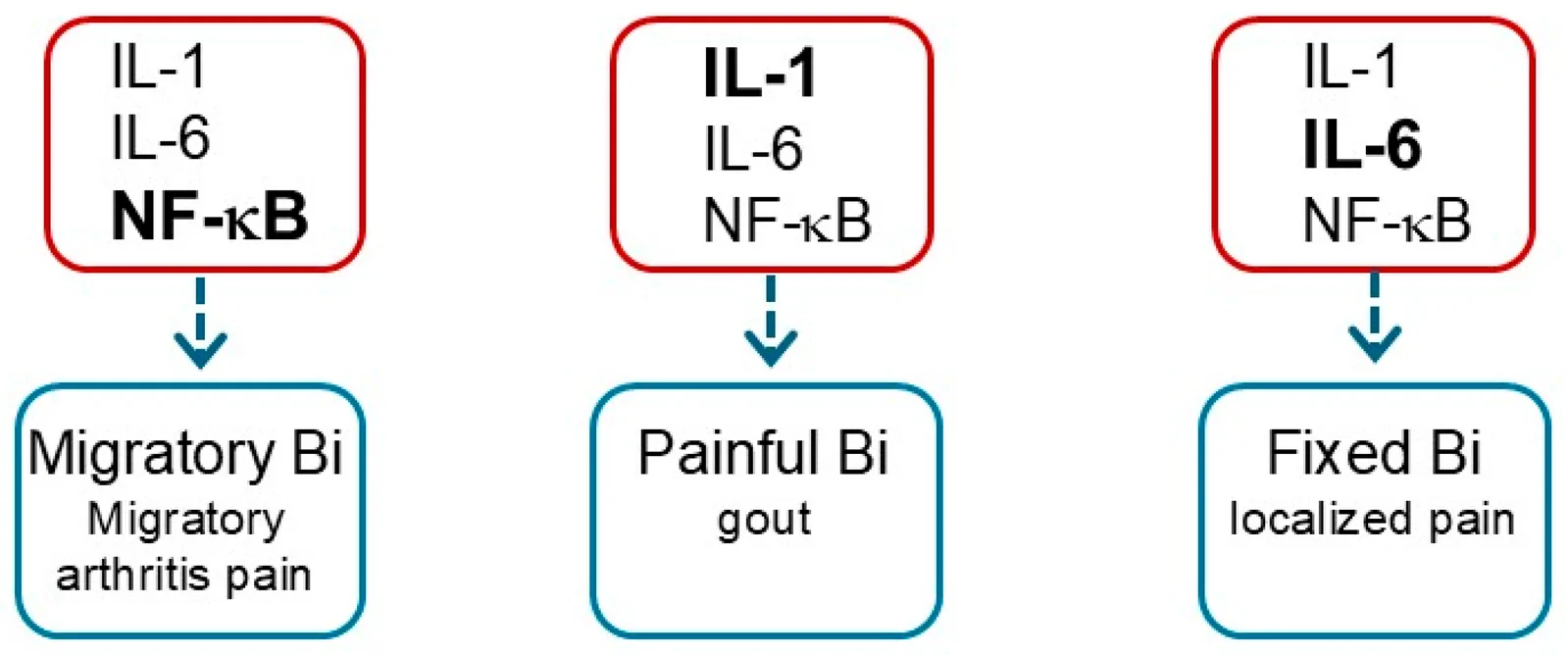
Theoretical mapping of inflammatory pathways to distinct Bi syndromes. According to the proposed YEIC framework, three primary inflammatory mediators—IL-1, IL-6, and NF-κB—are hypothesized to drive distinct phenotypic presentations of joint disease. Specifically, NF-κB predominance is theoretically mapped to Migratory Bi (characterized by migratory arthritis pain), IL-1 predominance to Painful Bi (gout), and IL-6 predominance to Fixed Bi (localized pain). IL, Interleukin; NF-κB, Nuclear factor kappa-light-chain-enhancer of activated B cells.

Figure 1 shows the relationship between inflammatory pathways and Bi syndromes: NF-κB produces “Migratory Bi,” IL-1 produces “Painful Bi” (gout), and IL-6 produces “Fixed Bi”.

### 3.2. NF-κB Induces Migratory Arthritis Pain

NF-κB plays a central role in rheumatoid arthritis by regulating inflammation, immune responses, synovial cell dysfunction, and osteoclast activation, with pathway blockade reducing disease severity in animal models [6]. In fibroblast-like synoviocytes (FLS), NF-κB activation drives proliferation, inhibits apoptosis, promotes inflammatory cytokine and matrix metalloproteinase production causing joint erosion, and enhances migration [7]. Importantly, FLS contribute to pain in osteoarthritis and rheumatoid arthritis by secreting inflammatory and pain mediators that interact with nerve and vascular endings, independent of structural damage [8]. NF-κB’s role in promoting the migratory behavior of synoviocytes is thus consistent with the migratory pattern of joint pain described in the YEIC, even if direct clinical evidence linking NF-κB to pain migration specifically remains to be established.

### 3.3. IL-1 Is Associated with Gout

IL-1 drives the inflammatory process in acute gout, making IL-1 inhibitors a promising treatment for refractory cases and patients intolerant to conventional therapies [9].

### 3.4. IL-6 Is Associated with Fixed Arthritis Pain

IL-6 promotes synovitis, joint destruction, and systemic inflammation in rheumatoid arthritis, with serum levels correlating directly with disease activity and severity [10].

### 3.5. Winter Bone Bi and Kidney Bi: YEIC Descriptions and Modern Medical Evidence

**Figure 2:**
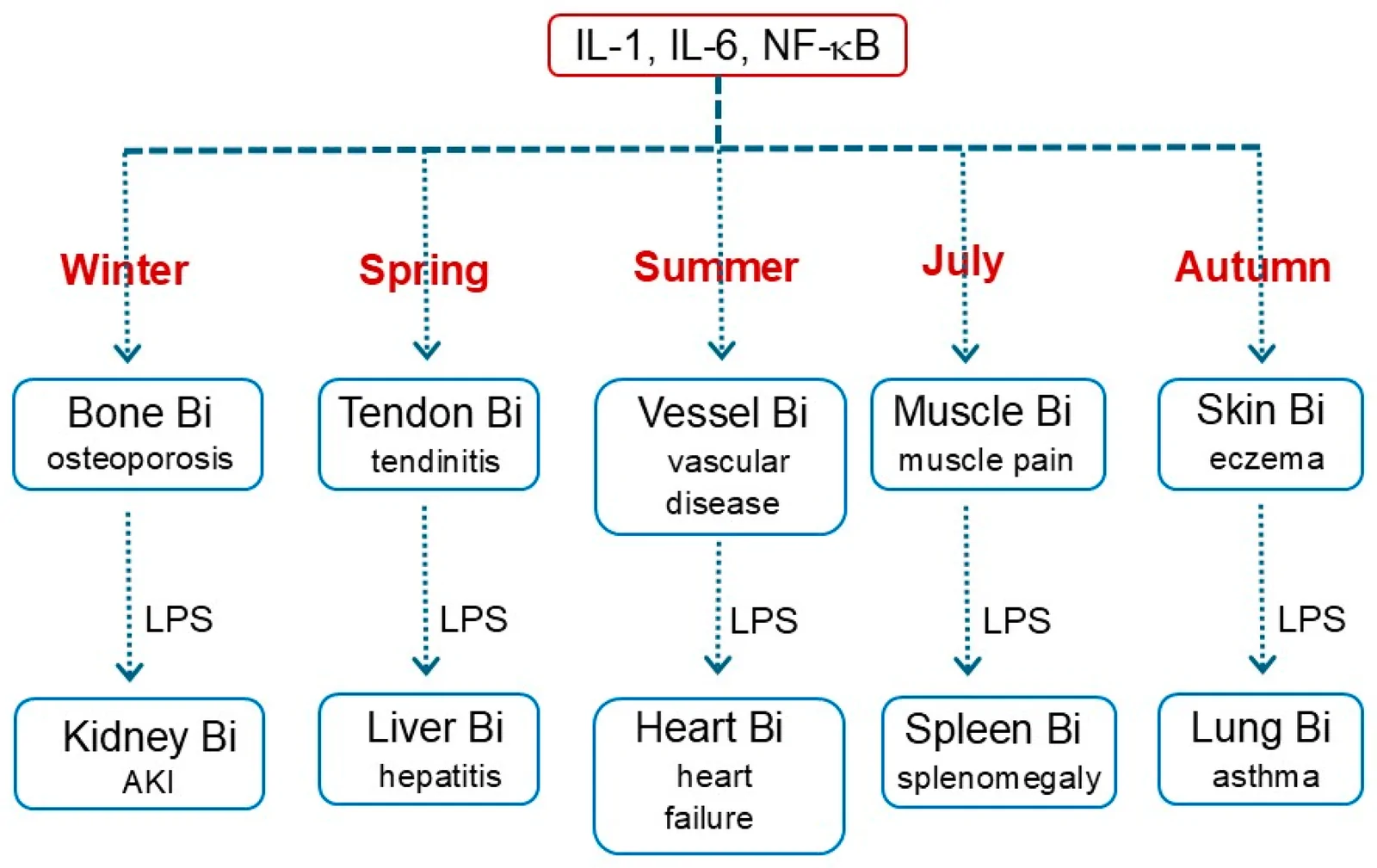
Proposed seasonal progression of Bi syndromes to organ injury under the YEIC framework. IL-1, IL-6, and NF-κB (top box) are proposed to predominate in five seasonally distinct Bi syndromes (arrows to season labels), each paired with a corresponding modern clinical correlate (e.g., Bone Bi/osteoporosis, winter); “July” denotes late summer. Persistent Bi syndrome is proposed to progress, via LPS exposure, to a corresponding visceral injury (e.g., Kidney Bi/acute kidney injury). All arrows depict theoretical progression pathways proposed within the YEIC framework, not established causal mechanisms (see Discussion for supporting and complicating evidence). Abbreviations: IL, interleukin; NF-κB, nuclear factor kappa-light-chain-enhancer of activated B cells; LPS, lipopolysaccharide; AKI, acute kidney injury. According to YEIC, IL-1, IL-6, and NF-κB cause “Bone Bi” in winter, characterized by bone heaviness, difficulty lifting limbs, severe marrow pain, and cold sensations. YEIC describes a pathological progression where persistent Bone Bi with concurrent LPS exposure allows pathogenic factors to invade the Kidneys, causing Kidney Bi with abdominal distension and severe kyphosis.

Figure 2 illustrates how IL-1, IL-6, and NF-κB drive distinct Bi syndromes in different seasons and shows disease evolution.

Modern evidence supports this framework. Winter is the peak season for bone disorders, with fragility fractures (hip, forearm, humerus, ankle) occurring most frequently in winter and least in summer [11,12,13,14]. Notably, this winter peak is not fully explained by icy or slippery conditions alone: hip fracture incidence shows a comparable winter peak even in Hong Kong, a subtropical setting without snow or ice, arguing against falls-related trauma as the sole explanation [14]. 

From a modern epidemiological perspective, this seasonal peak is conventionally attributed to reduced ambient temperature, lower UV-dependent vitamin D synthesis, and an increased propensity for falls during winter months. However, alongside these environmental factors, the inflammatory cascade mirrors the described pathology. Bone metabolism follows a circannual pattern with lower vitamin D, higher parathyroid hormone, and elevated bone turnover markers in winter, contributing to bone loss and osteoporosis over time [15,16,17,18]. For example, in a population-based cohort of women followed prospectively, spine bone mineral density was approximately 8.4% lower at its February nadir than at its August zenith, coinciding with the annual trough in serum vitamin D and peak in parathyroid hormone and bone resorption markers [17].

The inflammatory cascade mirrors the described pathology. Osteoporotic patients exhibit elevated monocyte IL-1 production correlating with bone turnover [19]. IL-1β and IL-6 promote bone loss and osteoporosis by stimulating osteoclast differentiation through RANKL upregulation, favoring bone resorption over formation [20,21]. NF-κB activation in osteoblasts inhibits bone formation, and its specific inhibition significantly prevents ovariectomy-induced bone loss [22]. 

Clinical manifestations align with YEIC descriptions. Low muscle mass associates with osteoporosis in both sexes [23], consistent with difficulty lifting limbs. Osteoporosis causes bone pain through increased sensory nerve fiber density, nociceptor sensitization from osteoclastic activity, and pathological nerve modifications [24]. Osteoporosis is associated with poor lower extremity circulation [25], correlating with cold sensations [26].

The progression to renal involvement aligns with seasonal patterns. Acute kidney injury (AKI) incidence peaks in winter across England, Japan, and China [27,28,29]; AKI is associated with increased fracture risk [30]. Supporting YEIC’s bone-kidney progression, ischemic AKI involves tubular epithelial cells generating proinflammatory cytokines including IL-6, IL-1β, and TNF-α that drive kidney injury [31]. NF-κB activation promotes renal inflammation, oxidative stress, and tissue injury in AKI pathogenesis [32]. LPS directly induces AKI through microcirculatory dysfunction, NLRP3 inflammasome activation, and mitochondrial damage [33].

Kidney Bi manifestations match precisely: chronic kidney disease-mineral and bone disorder can cause cervical kyphosis [34], and abdominal bloating is highly prevalent (45–50%) even in early CKD stages [35].

### 3.6. Spring Tendon Bi and Liver Bi: YEIC Descriptions and Modern Medical Evidence

As shown in Figure 2, IL-1, IL-6, and NF-κB cause “Tendon Bi” in spring, characterized by tendon spasms, joint pain, and difficulty walking. YEIC describes a pathological progression where persistent Tendon Bi with concurrent LPS exposure allows pathogenic factors to invade the Liver, causing Liver Bi with sleep disturbances, excessive thirst, frequent urination, and abdominal distention resembling pregnancy.

Modern evidence supports this framework. Tendinitis is a common injury during spring and summer [36]. The inflammatory cascade also mirrors the described pathology. IL-6 is elevated in human tendon tears, while IL-1β, IL-6, and TNF-α are increased in animal models of tendon injury [37]. IL-1β has been identified as a key mediator in tendinopathy pathogenesis, stimulating inflammation, apoptosis, and extracellular matrix degradation [38,39]. Transcriptional profiling of early rotator cuff tendinopathy reveals increased NF-κB signaling, which may drive degenerative changes prior to complete tears and impair healing following surgical repair [40].

Clinical manifestations align with YEIC descriptions. Tendon disease causes local pain, stiffness, and restricted movement [41], accompanied by protective muscle spasm around affected tendons [42], consistent with tendon spasms and difficulty walking.

The progression to hepatic involvement aligns with seasonal patterns. Hepatitis shows seasonal variation with spring and summer peaks for hepatitis A, B, C, and E [43], including acute hepatitis E in the UK [44]. Supporting YEIC’s tendon-liver progression, tendinitis may be the initial manifestation of autoimmune hepatitis [45], and chronic liver disease increases tendon disorder risk [46].

The cytokine-liver connection is well-established. IL-1 family cytokines, especially IL-1β, are central drivers of liver injury across viral, alcoholic, and metabolic hepatitis via inflammasome-dependent activation in hepatic macrophages [47,48]. Serum and intrahepatic IL-6 levels are elevated in chronic hepatitis B [49]. The NF-κB pathway in hepatocytes and immune cells orchestrates liver inflammatory responses in viral and autoimmune hepatitis by regulating cytokine expression [50,51]. LPS plays a central role in inflammatory liver disease pathogenesis [52].

Liver Bi manifestations match precisely: depression and anxiety are highly prevalent in hepatitis C patients [53], and 60–80% of chronic liver disease patients report poor sleep, including insomnia, reduced rapid eye movement (REM) sleep, and restless leg syndrome [54]. Persistent excessive thirst may signal serious liver disease [55]. Liver disease can damage kidneys, causing frequent urination [56], and liver disease frequently causes abdominal distention due to ascites [57].

### 3.7. Summer Vessel Bi and Heart Bi: YEIC Descriptions and Modern Medical Evidence

As shown in Figure 2, IL-1, IL-6, and NF-κB cause “Vessel Bi” in summer. Persistent Vessel Bi with concurrent LPS exposure progresses to Heart Bi, manifesting as poor circulation, palpitations with irritability, dyspnea, dry throat, belching, and anxiety. 

Modern evidence supports this framework. Each 1 °C rise above local temperature thresholds increases cardiovascular mortality by several percent, particularly for stroke and coronary heart disease [58]. Summer heat and humidity force the heart to nearly double blood circulation for thermoregulation [59]. Cold exposure, conversely, has also been linked to enhanced NF-κB activation and pro-inflammatory cytokine generation [60].

The inflammatory cascade mirrors the described pathology. Elevated IL-1β predicts worse outcomes in coronary artery disease and heart failure [61], while higher IL-6 increases risk for coronary disease, stroke, atrial fibrillation, and cardiovascular mortality [62,63]. NF-κB activation drives atherosclerotic plaque formation, endothelial dysfunction, myocardial hypertrophy, and heart failure progression [64,65,66]. Elevated circulating LPS correlates with carotid atherosclerosis progression [67].

The clinical presentation aligns precisely: cardiovascular disease causes poor circulation [26], irritability increases cardiovascular disease (CVD) risk [68], anxiety triggers palpitations [69], dyspnea links to asthma as a CVD risk factor [70], xerostomia commonly occurs in CVD patients [71,72], belching may indicate angina [73], and bidirectional fear-anxiety associations exist with heart disease [74,75]. Vascular disease and heart failure form a pathological cycle [76]. 

### 3.8. Late Summer (July) Muscle Bi and Spleen Bi: YEIC Descriptions and Modern Medical Evidence

As shown in Figure 2, IL-1, IL-6, and NF-κB trigger “Muscle Bi” in late summer (July), causing widespread muscular pain. Persistent Muscle Bi with concurrent LPS exposure progresses to Spleen Bi, manifesting as weakened limbs, coughing, vomiting, and dyspnea.

Modern evidence supports this framework. Muscle pain worsens in summer due to heat and dehydration [77]. The inflammatory cascade mirrors the described pathology: IL-1β from ischemia–reperfusion injury sensitizes muscle afferents and induces myalgia-like pain, which IL-1 receptor antagonists can block [78]. Intramuscular IL-6 produces dose-dependent mechanical hyperalgesia through gp130 signaling, inflammatory cell recruitment, cytokine production (TNF-α, IL-1β, KC/IL-8), and MAPK pathway activation [79]. NF-κB activation initiates transcription of inflammatory genes including TNF-α, IL-1β, COX-2, and IL-6 [80], with NF-κB-dependent mechanisms mediating inflammatory pain that pharmacological inhibition can block [81]. 

The progression to splenic involvement aligns with seasonal patterns. Juvenile birds exhibit significantly enlarged spleens in July [82], while human immune markers reach their lowest point in July: C-reactive protein and neutrophil counts reach their lowest levels, suggesting a compromised splenic immune response [83,84]. Supporting YEIC’s muscle-spleen progression, splenomegaly causes left upper abdominal pain radiating to the left shoulder [85,86]. 

The cytokine-spleen connection is well-established. Elevated IL-1β and IL-6 cause progressive splenomegaly [87], increased NF-κB activation drives splenic inflammation and enlargement [88], and LPS triggers splenomegaly through inflammatory activation [89,90].

Clinical manifestations align precisely: splenomegaly produces anemia-related weakness, fatigue, and exertional intolerance perceived as limb weakness [91], presents with vomiting in hepatosplenomegaly [92], and causes dyspnea from underlying anemia [93]. 

### 3.9. Autumn Skin Bi and Lung Bi: YEIC Descriptions and Modern Medical Evidence

As shown in Figure 2, IL-1, IL-6 and NF-κB cause “Skin Bi” in autumn. YEIC describes a pathological progression where persistent Skin Bi with concurrent LPS exposure allows pathogenic factors to invade the Lungs, resulting in Lung Bi, manifesting as anxiety, chest tightness, dyspnea, and vomiting.

Modern evidence supports this framework. Eczema worsens in autumn [94]. Atopic dermatitis shows upregulated IL-1β and IL-18, promoting cutaneous inflammation [95], with elevated IL-1β and IL-6 in lesions [96]. NF-κB-DNA binding activity is significantly higher in eczema patients and correlates with clinical severity [97].

The skin-lung progression aligns with seasonal patterns. The “September asthma peak” shows 2–3-fold increases in emergency visits versus summer [98]. Atopic dermatitis patients have increased asthma risk through shared Th2-mediated inflammatory pathways [99].

The cytokine-lung connection is well-established. Asthma shows elevated IL-1β and IL-6 mediating airway inflammation [100], with NF-κB playing a central role [101]. LPS exposure increases asthma severity: elevated airway LPS correlates with neutrophil recruitment and airway remodeling [102], while potentiating allergen-induced inflammation even in corticosteroid-treated patients [103].

Clinical manifestations align precisely: anxiety and depression link to poor asthma control [104], airway inflammation produces chest tightness and dyspnea [105], and vomiting can be a dominant symptom of acute asthma in children [106].

## 4. Discussion

This review was undertaken to examine whether descriptions in the Yellow Emperor’s Inner Canon (YEIC) of seasonal disease patterns and Bi syndrome could be systematically mapped onto three well-characterized inflammatory mediators, IL-1, IL-6, and NF-κB, and whether the resulting framework aligns with independently documented evidence in the biomedical literature. Across the Bi syndromes and the seasonal organ-progression pattern examined here, we found consistent thematic and temporal correspondence between the YEIC’s classical descriptions and modern findings concerning these three mediators. As detailed below, this correspondence should be understood as a set of biologically plausible, hypothesis-generating associations rather than as evidence of an established causal mechanism.

In the YEIC, the symptoms arising from the combined actions of IL-1, IL-6, and NF-κB are collectively termed “Bi” syndrome. This condition is further designated by the affected anatomical region. When each of the three molecules exerts a predominant influence, the condition is classified according to its distinct pathological characteristics as Painful Bi, Fixed Bi, or Migratory Bi, respectively.

Within the theoretical framework of the YEIC, “Wind” corresponds to NF-κB [2]. Since wind is by nature unpredictable and wandering, NF-κB predominance manifests clinically as migratory pain. Accordingly, NF-κB is identified as the principal driver of migratory joint pain [2].

Likewise, the YEIC maps “Cold” onto IL-1. When IL-1 predominates, clinical presentation is one of severe pain, implicating IL-1 as the primary pathological basis of gout [2].

The YEIC associates “Dampness” with IL-6. Dampness is adhesive in nature, tending to accumulate and stagnate in a fixed location. When IL-6 predominates, this manifests as localized, fixed pain—the hallmark of Fixed Bi [2].

The YEIC delineates the season-dependent, tissue-specific effects of IL-1, IL-6, and NF-κB on the human body.

In winter, these three molecules preferentially target bone tissue, manifesting in conditions such as osteoporosis. If left untreated, the concurrent influence of LPS drives further pathological progression to the kidneys, potentially culminating in acute kidney injury (AKI).

In spring, the primary targets shift to the tendons and sinews, where dysregulation of these molecules can give rise to tendinitis. Without timely intervention, LPS potentiates the spread of pathology to the liver, precipitating hepatitis.

In summer, the three molecules predominantly affect the vascular system, contributing to a spectrum of vascular diseases. Should the condition remain unaddressed, LPS-mediated amplification can extend the damage to the heart, ultimately resulting in heart failure.

In July—referred to in the YEIC as the “Late Summer”—the molecular targets migrate to skeletal muscle, manifesting as myalgia and related muscular disorders. In the absence of effective treatment, LPS-driven progression can lead to splenic involvement, causing splenomegaly.

In autumn, these molecules act principally upon the skin, where their dysregulation underlies conditions such as eczema. If inadequately managed, LPS further propagates the inflammatory cascade to the lungs, predisposing individuals to asthma.

These seasonal pathological patterns align well with observations documented in contemporary medical literature, yet modern medicine tends to treat each condition as an isolated entity, overlooking the unified pathophysiological framework that the YEIC had already articulated millennia ago.

Although modern medicine has observed and documented these seasonal phenomena, it has yet to recognize the season-dependent differential roles played by IL-1, IL-6, and NF-κB in driving them. Instead, it has consistently attributed the seasonal patterns of these conditions to non-medical factors, dismissing them with behavioral or environmental explanations. Tendon injuries serve as a representative example: spring marks the peak season for tendon injuries, with the incidence of Achilles tendon ruptures rising in a statistically significant manner compared to autumn and winter—a trend especially pronounced in sport-related cases. The explanation conventionally offered by modern medicine is that individuals who have experienced physical deconditioning over the winter months return in large numbers to outdoor activities as temperatures rise, resulting in a sudden increase in mechanical load on the tendons [107,108]. This account, however, is confined to behavioral causation and entirely overlooks the underlying season-dependent molecular biological mechanisms—which may, in fact, represent the more fundamental basis for understanding this pattern.

A clarification is warranted here. Taking winter as an example, the concept of “Bone Bi” described in the YEIC does not exclusively correspond to osteoporosis as defined in modern medicine. Rather, it serves as a broad designation encompassing the full spectrum of skeletal disorders arising from the aberrant activation of IL-1, IL-6, and NF-κB; osteoporosis represents merely one of its many clinical manifestations. This interpretive principle applies equally to the prevalent conditions associated with other seasons—the specific diseases cited for each season should be understood as representative examples of that season’s characteristic pathological tendencies, not as a complete list.

The general premise that inflammatory activity varies by season is independently supported by the broader chronoimmunology literature. Using genome-wide transcriptional profiling unrelated to any traditional medicine framework, Dopico et al. found that the human immune system exhibits a pro-inflammatory gene-expression profile during the European winter, including elevated soluble IL-6 receptor and C-reactive protein, coinciding with the peak incidence of several cardiovascular, psychiatric, and autoimmune conditions [5]. This independent line of evidence supports the general phenomenon underlying the present analysis: that winter is associated with heightened inflammatory activity. The contribution of the present study is narrower and more specific than this existing literature: rather than establishing that inflammation varies seasonally in general, we propose that particular mediators correspond to particular YEIC-described syndromes and organ-progression patterns. To our knowledge, this more granular correspondence has not been directly tested in the chronoimmunology literature and remains to be validated.

Several limitations of the present analysis warrant acknowledgment. The evidence linking cytokine activity to seasonal patterns is largely correlational. While the seasonal epidemiology of each condition cited is well-documented in the literature, the mechanisms underlying these seasonal rhythms—and the specific contributions of IL-1, IL-6, and NF-κB to their timing—have yet to be established through prospective studies designed for this purpose. The seasonal associations described here are therefore hypothesis-generating rather than mechanistically conclusive, pointing toward a testable question: whether the inflammatory mechanisms now characterized in modern biomedicine are the biological basis of seasonal patterns that physicians documented two thousand years ago. 

A further complication concerns the seasonal timing proposed for Vessel Bi and Heart Bi. The present analysis proposes that IL-1, IL-6, and NF-κB predominantly drive vascular and cardiac pathology in summer, citing heat-related increases in cardiovascular mortality above local temperature thresholds in support [58]. This specific mechanism is corroborated by subsequent evidence: a county-level analysis across the contiguous United States found extreme heat directly associated with excess cardiovascular mortality concentrated in summer months [109]. This heat-related effect, however, represents a comparatively narrow phenomenon within a much larger and more consistently documented pattern: across many countries and climates, cardiovascular disease of nearly all subtypes—including myocardial infarction, stroke, and heart failure—shows a pronounced winter peak, with event rates typically 10–20% higher in winter than in summer [110]. This winter predominance, rather than a summer predominance, remains the dominant seasonal signal in the cardiovascular epidemiology literature considered as a whole. Taken together, these findings present a genuinely mixed picture: the summer-specific, heat-driven cardiovascular mechanism proposed here is real and independently documented, lending partial support to the YEIC’s seasonal claim, but it operates alongside, and appears quantitatively smaller than, an opposing winter-predominant pattern likely driven by cold-related physiological stress. This does not undermine the plausibility of IL-1, IL-6, and NF-κB as contributors to cardiovascular pathology—each is independently well supported—but it does indicate that the vascular and cardiac vulnerability the YEIC associates with summer reflects one of two distinct, opposing seasonal mechanisms—heat-driven in summer and cold-driven in winter—with the latter currently the more extensively documented contributor to overall cardiovascular event rates.

Furthermore, the search strategy was designed to identify literature supporting the YEIC framework, which inherently introduces confirmation bias. It is crucial to acknowledge competing explanations for seasonal disease variations, including circadian and circannual immunology—encompassing photoperiod-driven and endogenous rhythms in immune cell trafficking and cytokine production that operate independently of any traditional medicine framework [5]—circannual variation in vitamin D, ambient temperature and humidity fluctuations, infectious exposure cycles, seasonal aeroallergen (e.g., pollen) exposure, changes in physical activity, and socioeconomic factors such as differential healthcare-seeking behavior and diagnostic reporting across seasons. The molecular mappings utilized in this study (e.g., Cold to IL-1, Dampness to IL-6) are derived from previous theoretical work and serve as the premise of this investigation, but they are not yet universally established biomedical relationships. Therefore, the framework presented here should be viewed strictly as hypothesis-generating. These limitations notwithstanding, the degree of concordance between the YEIC framework and contemporary biomedical observations is sufficient to warrant further systematic investigation.

IL-1, IL-6, and NF-κB are proposed here as a shared explanatory thread across several conditions—osteoporosis, tendinopathy, vascular disease, myalgia, and eczema—that are not conventionally understood to share a common mechanism. This analysis does not claim that these three mediators are the best available predictors of seasonal timing for any single condition in isolation; well-established, condition-specific explanations exist for several of the domains considered (e.g., the vitamin D–parathyroid hormone axis for winter bone loss, mechanical reconditioning for spring tendon injury) and are not disputed here. The relevant comparison for this framework’s contribution is therefore not single-condition predictive superiority, but whether a shared three-mediator pathway offers a more parsimonious account of the co-occurrence of these conditions than the current disease-specific literature, in which no single alternative factor—temperature, vitamin D, infection cycles, or behavior—is proposed as a common mechanism across all five domains. This analysis does not formally test that comparison; doing so would require a study assessing whether cytokine measures explain shared variance across multiple disease outcomes within one cohort, beyond what these covariates explain individually. This is a necessary direction for future work.

## 5. Conclusions

Since its compilation more than two millennia ago, the Yellow Emperor’s Inner Canon (YEIC) has remained a subject of enduring debate. Over the past two centuries, the rise of modern biomedicine led Western medicine to adopt a largely dismissive stance toward the text, while skepticism persisted even within Traditional Chinese Medicine (TCM) circles. Recent advances in molecular biology—together with emerging candidate correspondences between Vital TCM Substances such as Qi, Cold, and Dampness and specific molecular mechanisms—now provide an opportunity to re-examine this classical work through a contemporary scientific lens.

The present analysis suggests that several YEIC concepts may exhibit thematic parallels with modern cytokine biology. These parallels should be interpreted as hypothesis-generating observations rather than demonstrated equivalences. Rigorous, prospective studies will be required to determine whether these correspondences reflect genuine underlying biology or merely retrospective pattern-matching.

With respect to Bi syndrome, the central proposal of this interpretive framework may be summarized as follows: NF-κB predominance is proposed to correspond to migratory joint pain; IL-1 predominance to gout; and IL-6 predominance to fixed-location joint pain. These associations arise from conceptual parallels between YEIC descriptions and known cytokine functions. They have not been tested experimentally and should therefore be regarded as provisional rather than confirmed mechanisms.

The YEIC further describes a seasonal progression in which the primary target organ of pathological activity shifts across the year. Within this framework, winter is associated with bone-related conditions such as osteoporosis, with potential progression to kidney involvement; spring with tendon and sinew disorders such as tendinitis, with potential progression to liver involvement; summer with vascular disorders, with possible progression to heart involvement (though independent epidemiological evidence identifies winter, rather than summer, as the more consistently documented peak season for overall cardiovascular event rates); late summer (approximately July) with muscle-related conditions such as myalgia, with potential progression to splenic involvement; and autumn with skin disorders such as eczema, with potential progression to pulmonary involvement. The individual seasonal associations cited here are supported by epidemiological literature. However, the proposed unifying role of IL-1, IL-6, and NF-κB across this seasonal cycle remains a theoretical construct rather than an established biological mechanism.

Modern biomedicine typically regards skeletal disease, acute kidney injury, tendinopathy, hepatitis, vascular disease, heart disease, myalgia, splenomegaly, eczema, and asthma as largely distinct entities. In contrast, the YEIC offers a unifying interpretive framework in which seasonal variation in IL-1, IL-6, and NF-κB activity may contribute to the onset and progression of each. Three tiers of evidence should be distinguished when evaluating this proposal. First, the seasonal epidemiological patterns of these conditions are observed associations well documented in the literature. Second, the involvement of IL-1, IL-6, and NF-κB in the underlying pathology of most of these conditions is biologically plausible and independently supported by mechanistic studies. Third, the specific claim that seasonal variation in these mediators constitutes a common driver linking all of these conditions within a single YEIC-derived framework has not been established and remains speculative.

The parallels identified here between a classical medical text and contemporary cytokine biology merit further investigation, but they should be interpreted with appropriate caution until validated by prospective, mechanistic, and controlled studies designed explicitly to test them. This article provides only an introductory exploration of Bi syndrome; future work must evaluate—rather than assume—the proposed associations before they can inform any therapeutic strategy. Should this shared mechanism withstand such validation, it may offer a basis for reconsidering conventionally segregated inflammatory conditions as sharing overlapping, rather than isolated, seasonal pathophysiology.

## Figures and Tables

**Table 1:** The mapping between TCM vital substances and signaling molecules.

TCM Vital Substance	Signaling Molecules	TCM Vital Substance	Signaling Molecules
Essence	Tyrosine	Liver Qi	Glutathione (GSH)
Mind	Melatonin	Liver Yin	Superoxide dismutase (SOD) Renin
Ethereal Soul	Serotonin	Liver Yang	Hepatocyte growth factor (HGF) Erythropoietin (EPO)
Corporeal Soul	Cysteine	Heart Qi	Magnesium (Mg)
Ideation	Tryptophan	Heart Yin	Angiotensin
Will	Arginine	Heart Yang	IGF
Thin Fluid	Proteins	Spleen	Ghrelin
Thick Fluid	Lipids	Spleen Yin	Aldosterone Retinoic Acid
Qi	Sodium	Spleen Yang	Atrial Natriuretic Peptide (ANP)
Zong Qi	Dopamine	Lung Qi	Hypoxia-Inducible Factor (HIF)
Nutrient Qi	Cyclic adenosine monophosphate (cAMP)	Lung Yin	Ascorbic Acid
Glory Qi	Adenosine triphosphate (ATP)	Lung Yang	Vascular endothelial growth factor (VEGF) Fibroblast Growth Factor 7 (FGF7)
Defending Qi	Chloride	Kidney Qi	Nitric Oxide (NO)
Yin Qi	Potassium	Kidney Yin	Wnt
Yang Qi	Calcium	Kidney Yang	Calcitonin Parathyroid hormone-related protein (PTHrP)
Clear Qi	Toll-like receptor 4 (TLR4)	Cold	Interleukin-1 (IL-1)
Turbid Qi	Leptin	Damp	Interleukin-6 (IL-6)
Righteous Qi	Immunoglobulin G (IgG)	Wind	NF-κB
Evil Qi	Lipopolysaccharide (LPS)	Heat	TNF-α
Stomach Qi	Amylase	Dry	Interleukin-8 (IL-8)
Genuine Qi	Phosphorus	Summerheat	Cyclooxygenase-2 (COX-2)
Blood Qi	Iron	Consumption	Forkhead Box O1 (FoxO1)
Essence Qi	Zinc	Central	Aldosterone
Indulged Qi	Gonadotropin-releasing hormone (GnRH)	Mind Qi	Thyroid Hormone

## Data Availability

No new data were created or analyzed in this study. Data sharing is not applicable to this article.
